# Publisher Correction: Fibrillin microfibril structure identifies long-range effects of inherited pathogenic mutations affecting a key regulatory latent TGFβ-binding site

**DOI:** 10.1038/s41594-023-01050-3

**Published:** 2023-06-30

**Authors:** Alan R. F. Godwin, Rana Dajani, Xinyang Zhang, Jennifer Thomson, David F. Holmes, Christin S. Adamo, Gerhard Sengle, Michael J. Sherratt, Alan M. Roseman, Clair Baldock

**Affiliations:** 1 Wellcome Trust Centre for Cell-Matrix Research, Division of Cell Matrix Biology and Regenerative Medicine, School of Biological Sciences, Faculty of Biology, Medicine and Health, University of Manchester, Manchester Academic Health Science Centre, Manchester, UK; 2 Center for Biochemistry, Faculty of Medicine and University Hospital Cologne, University of Cologne, Cologne, Germany; 3 Department of Pediatrics and Adolescent Medicine, Faculty of Medicine and University Hospital Cologne, University of Cologne, Cologne, Germany; 4 Center for Molecular Medicine Cologne, University of Cologne, Cologne, Germany; 5 Cologne Center for Musculoskeletal Biomechanics, Cologne, Germany; 6 Division of Cell Matrix Biology and Regenerative Medicine, School of Biological Sciences, Faculty of Biology, Medicine and Health, University of Manchester, Manchester Academic Health Science Centre, Manchester, UK; 7 Division of Molecular and Cellular Function, School of Biological Sciences, Faculty of Biology, Medicine and Health, University of Manchester, Manchester Academic Health Science Centre, Manchester, UK

**Keywords:** Cryoelectron microscopy, Supramolecular assembly, Structure determination, Cryoelectron microscopy, Cell growth

Correction to: *Nature Structural & Molecular Biology* 10.1038/s41594-023-00950-8. Published online 20 April 2023.

In the version of this article initially published, in Figure 4a, the top panel (PF3) was inadvertently duplicated in the bottom panel (PF3 WMS). This caused the wrong data to be displayed for the PF3 WMS analysis. The error has been corrected in the HTML and PDF versions of the article.

