# Supplementary material for: Fibrillin microfibril structure identifies long-range effects of inherited pathogenic mutations affecting a key regulatory latent TGFβ-binding site
Source: Nat Struct Mol Biol. 2023 Apr 20;30(5):608–18. doi: 10.1038/s41594-023-00950-8 (PMC10191836; doi:10.1038/s41594-023-00950-8)
Supplement: Supplementary file 1 — Reporting Summary [file 41594_2023_950_MOESM1_ESM.pdf]

nature portfolio

Corresponding author(s): Clair Baldock

Last updated by author(s): Jan 21, 2023

# Reporting Summary

Nature Portfolio wishes to improve the reproducibility of the work that we publish. This form provides structure for consistency and transparency in reporting. For further information on Nature Portfolio policies, see our Editorial Policies and the Editorial Policy Checklist.

## Statistics

For all statistical analyses, confirm that the following items are present in the figure legend, table legend, main text, or Methods section.

| n/a | Confirmed | |
|---|---|---|
| ☐ | ☒ | The exact sample size (*n*) for each experimental group/condition, given as a discrete number and unit of measurement |
| ☐ | ☒ | A statement on whether measurements were taken from distinct samples or whether the same sample was measured repeatedly |
| ☐ | ☒ | The statistical test(s) used AND whether they are one- or two-sided<br>*Only common tests should be described solely by name; describe more complex techniques in the Methods section.* |
| ☐ | ☒ | A description of all covariates tested |
| ☐ | ☒ | A description of any assumptions or corrections, such as tests of normality and adjustment for multiple comparisons |
| ☐ | ☒ | A full description of the statistical parameters including central tendency (e.g. means) or other basic estimates (e.g. regression coefficient) AND variation (e.g. standard deviation) or associated estimates of uncertainty (e.g. confidence intervals) |
| ☐ | ☒ | For null hypothesis testing, the test statistic (e.g. *F*, *t*, *r*) with confidence intervals, effect sizes, degrees of freedom and *P* value noted<br>*Give P values as exact values whenever suitable.* |
| ☒ | ☐ | For Bayesian analysis, information on the choice of priors and Markov chain Monte Carlo settings |
| ☒ | ☐ | For hierarchical and complex designs, identification of the appropriate level for tests and full reporting of outcomes |
| ☒ | ☐ | Estimates of effect sizes (e.g. Cohen's *d*, Pearson's *r*), indicating how they were calculated |

*Our web collection on statistics for biologists contains articles on many of the points above.*

## Software and code

Policy information about availability of computer code

| | |
|---|---|
| Data collection | CryoEM data was collected on a Titan Krios electron microscope, data collection was automated by the Thermofisher EPU (v1.9) software. |
| Data analysis | CryoEM data analysis utilised Relion2.1, MotionCor2 v1.2.0, Warp 1.0.4, cryoSPARC v2. Movies were motion corrected and dose weighted using MotionCor2 v1.2.0. Corrected images were imported into Warp 1.0.4 where particles were picked using a Warp box net. Particle stacks were imported into cryoSPARC v2 and were used in a homogenous refinement. The resulting structure from cryoSPARC v2 was then further refined in RELION 2.1. SPIDER (version UNIX 26.04) was used for symmetry analysis. Models of cryoEM structures were visualised in UCSF ChimeraX 1.13.1rc.<br><br>Negative stain TEM: The periodicity of microfibril repeats was measured using ImageJ2.<br><br>STEM mass mapping: Mass per unit length measurements and axial mass distributions were measured from STEM ADF images using the Semper6 image analysis software (Synoptics).<br><br>STEM mass data and periodicity measurements were analysed in Graphpad Prism 9.1.2. |

For manuscripts utilizing custom algorithms or software that are central to the research but not yet described in published literature, software must be made available to editors and reviewers. We strongly encourage code deposition in a community repository (e.g. GitHub). See the Nature Portfolio guidelines for submitting code & software for further information.

## Data

Policy information about availability of data

All manuscripts must include a data availability statement. This statement should provide the following information, where applicable:

- Accession codes, unique identifiers, or web links for publicly available datasets
- A description of any restrictions on data availability
- For clinical datasets or third party data, please ensure that the statement adheres to our policy

The cryoEM data has been deposited to EMBD with accession codes EMD-13984 for the bead model and EMD-13986 for the arm region. The values plotted in figures 3 and 4 are provided as source data online. Any additional information required to reanalyse the data reported in this study is available from the corresponding author.

# Field-specific reporting

Please select the one below that is the best fit for your research. If you are not sure, read the appropriate sections before making your selection.

☒ Life sciences ☐ Behavioural & social sciences ☐ Ecological, evolutionary & environmental sciences

For a reference copy of the document with all sections, see nature.com/documents/nr-reporting-summary-flat.pdf

# Life sciences study design

All studies must disclose on these points even when the disclosure is negative.

| | |
|---|---|
| Sample size | No sample size calculations were performed. For cryoEM imaging of bovine microfibrils, at least six independent datasets were collected in the optimisation of the sample and data collection strategy. For the final cryoEM dataset, 1310 movies were collected and 27,737 microfibril periods were picked from the motion corrected images.<br><br>For negative stain or STEM mass mapping of microfibrils, 100 periods were measured. We determined this to be sufficient based on previous similar studies and low variability between samples doi: 10.1016/j.jmb.2010.04.008; doi: 10.1016/s1357-2725(97)00028-9.<br><br>Biochemical experiments were repeated at least three times, with each experiment containing thousands of molecules. Performing biochemical experiments in biological triplicate is a widely used replication standard. |
| Data exclusions | The cryoEM pipeline excludes “bad” particles in an automated software pipeline that is widely utilised in the field and is well-described.<br>For all other data types, no data were excluded from analysis. |
| Replication | All attempts at replication were successful.<br>Biochemical experiments were repeated at least three times.<br>Negative staining imaging on wildtype and mutant microfibrils was repeated at least twice on each different tissue sample.<br>For cryoEM, more than six microfibril purifications were performed on six different ciliary zonule samples (biological replicates) for optimisation of grid preparation and data collection strategy. At least six cryoEM datasets were collected independently in the optimisation process and all data were consistent with the final data set. |
| Randomization | Randomization is not relevant for this study, as there were no groups allocated in any of the experiments. |
| Blinding | Blinding was not relevant for the cryoEM data as data was collected on only one sample type.<br>For STEM mass mapping and negative stain periodicity measurements, blinding was not performed as in each sample, images of all microfibrils were collected and from these images all microfibril periods were included. The same measurement procedures were used for all images and for the negative stain data particle picking and classification was automated to prevent bias. The readout from these data was a quantifiable parameter ie mass per repeat or microfibril periodicity, respectively, rather than a subjective observation. |

# Reporting for specific materials, systems and methods

We require information from authors about some types of materials, experimental systems and methods used in many studies. Here, indicate whether each material, system or method listed is relevant to your study. If you are not sure if a list item applies to your research, read the appropriate section before selecting a response.

## Materials & experimental systems

| n/a | Involved in the study |
| --- | --- |
| ☐ | ☒ Antibodies |
| ☒ | ☐ Eukaryotic cell lines |
| ☒ | ☐ Palaeontology and archaeology |
| ☐ | ☒ Animals and other organisms |
| ☒ | ☐ Human research participants |
| ☒ | ☐ Clinical data |
| ☒ | ☐ Dual use research of concern |

## Methods

| n/a | Involved in the study |
| --- | --- |
| ☒ | ☐ ChIP-seq |
| ☒ | ☐ Flow cytometry |
| ☒ | ☐ MRI-based neuroimaging |

# Antibodies

| | |
| --- | --- |
| Antibodies used | Commercial antibodies from Sigma Aldrich MAB2502 (Anti-Fibrillin-1 Antibody, clone 26; Lot number 0608038538) and MAB1919 (Anti-Fibrillin-1 Antibody, clone 11C1.3; Lot number 3256666 ). |
| Validation | MAB2502 recognizes human Fibrillin-1. Epitope mapping studies identify the binding site of this antibody to amino-terminal end of the molecule, between amino acid residues 45 and 450. The antibody is reactive with human, chicken, and bovine Fibrillin-1.<br>MAB1919 recognizes human Fibrillin-1. The antibody is reactive with bovine, pig, and human Fibrillin-1.<br>Publications DOI: 10.1242/jcs.029819, DOI: 10.1016/0945-053x(94)90028-0; DOI: 10.1074/jbc.M111.231571; DOI: 10.1006/jmbi.1996.0237<br>In the manuscript, both antibodies are used in western blotting against human fibrillin-1 constructs to further define their epitopes to map their binding location on fiibrilln microfibrils. |

# Animals and other organisms

Policy information about studies involving animals; ARRIVE guidelines recommended for reporting animal research

| | |
| --- | --- |
| Laboratory animals | Skin samples were collected from 6-week old male homozygous WMΔ mice, ΔHybrid1 mice or control (C57BL/6) mice |
| Wild animals | The study did not involve wild animals |
| Field-collected samples | The study did not involve samples collected from the field |
| Ethics oversight | Landesamt für Natur, Umwelt und Verbraucherschutz Nordrhein-Westfalen for breeding (permit No. 84-02.04.2014.A397) and euthanasia (permit No. 84-02.05.40.14.115). |

Note that full information on the approval of the study protocol must also be provided in the manuscript.
